# Atherosclerosis severity is not affected by a deficiency in IL‐33/ST2 signaling

**DOI:** 10.1002/iid3.62

**Published:** 2015-05-10

**Authors:** Praxedis Martin, Gaby Palmer, Emiliana Rodriguez, Estelle Woldt, Isabelle Mean, Richard W. James, Dirk E. Smith, Brenda R. Kwak, Cem Gabay

**Affiliations:** ^1^ Division of RheumatologyUniversity Hospital of GenevaGenevaSwitzerland; ^2^ Department of Pathology and ImmunologySchool of MedicineUniversity of GenevaGenevaSwitzerland; ^3^ Division of Diabetes, Endocrinology and NutritionDepartment of Internal MedicineUniversity Hospital of GenevaGenevaSwitzerland; ^4^ Inflammation Research DepartmentAmgen Inc.SeattleWashingtonUSA; ^5^ Division of CardiologyUniversity Hospital of GenevaGenevaSwitzerland

**Keywords:** Atherosclerosis, IL‐33, ST2, Th1‐to‐Th2‐shift

## Abstract

Interleukin (IL)‐33 is a cytokine of the IL‐1 family, which signals through the ST2 receptor. Previous work demonstrated that the systemic administration of recombinant IL‐33 reduces the development of atherosclerosis in apolipoprotein E‐deficient (ApoE^−/−^) mice by inducing a Th1‐to‐Th2 shift. The objective of our study was to examine the role of endogenous IL‐33 and ST2 in atherosclerosis. ApoE^−/−^, IL‐33^−/−^ApoE^−/−^, and ST2^−/−^ApoE^−/−^ mice were fed with a cholesterol‐rich diet for 10 weeks. Additionally, a group of ApoE^−/−^ mice was injected with a neutralizing anti‐ST2 or an isotype control antibody during the period of the cholesterol‐rich diet. Atherosclerotic lesion development was measured by Oil Red O staining in the thoracic‐abdominal aorta and the aortic sinus. There were no significant differences in the lipid‐staining area of IL‐33^−/−^ApoE^−/−^, ST2^−/−^ApoE^−/−^, or anti‐ST2 antibody‐treated ApoE^−/−^ mice, compared to ApoE^−/−^ controls. The absence of IL‐33 signaling had no major and consistent impact on the Th1/Th2 cytokine responses in the supernatant of in vitro‐stimulated lymph node cells. In summary, deficiency of the endogenously produced IL‐33 and its receptor ST2 does not impact the development of atherosclerosis in ApoE‐deficient mice.

## Introduction

Interleukin (IL)‐33 is the most recently discovered member of the IL‐1 cytokine family (see 1 for review). As a dual function protein IL‐33 displays both intracellular and extracellular effects. The extracellular effect is executed by binding to its receptor T1/ST2 (ST2), a member of the IL‐1 receptor family 2. IL‐33 is mainly expressed as a nuclear protein in stromal cells, including specialized fibroblasts, epithelial, and human endothelial cells, but only to a limited extent in mouse endothelial cells 3, 4. Upon cell damage during trauma or infection, IL‐33 can be released from necrotic cells and act as an alarmin to alert the immune system 3. ST2 is predominantly expressed on type 2 innate lymphoid cells, activated Th2 lymphocytes, and mast cells 5, 6, 7, but its expression has also been demonstrated on basophils, eosinophils, and dendritic cells. IL‐33 induces the production of Th2 cytokines, enhances serum immunoglobulin synthesis and is, therefore, associated with Th2‐dependent inflammatory diseases 2.

Atherosclerosis is a chronic inflammatory disease characterized by the formation of arterial lesions, which consist of infiltrating T cells, macrophages that convert into foam cells, as well as resident smooth muscle and endothelial cells producing cytokines, growth factors, and other pro‐inflammatory mediators 8. Several studies provide evidence for a protective or beneficial role of IL‐33 in cardiovascular biology 9, 10. Of note, the administration of recombinant IL‐33 exerted a protective effect in the apolipoprotein E‐deficient (ApoE^‐/‐^) mouse model of atherosclerosis. IL‐33 treatment reduced the lesion size in the aortic sinus and induced a Th1‐to‐Th2 switch by increasing the levels of the Th2 cytokines IL‐4, IL‐5, and IL‐13 and decreasing the Th1 cytokine IFNγ in serum and lymph node cells. Coadministration of a neutralizing anti‐IL‐5 antibody with IL‐33 prevented the reduction in plaque size, indicating that IL‐5 mediated the atheroprotective effect of IL‐33. The neutralization of endogenous IL‐33 by the administration of soluble ST2, which acts as a decoy receptor for IL‐33, exacerbated the development of atherosclerotic plaques in the aortic sinus and led to increased IFNγ levels in both serum and the supernatant of in vitro‐stimulated peripheral lymph node cells 11. These data suggested that exogenously administrated IL‐33 plays a protective role in the development of atherosclerosis. However, no study addressed the development of atherosclerosis in atherosclerotic‐prone mice deficient in either IL‐33 or ST2. Therefore, we generated IL‐33^−/−^ApoE^−/−^ and ST2^−/−^ApoE^−/−^ mice for a side‐by‐side analysis and in addition, treated ApoE^−/−^ mice with a blocking anti‐ST2 or the isotype‐matched control antibody in order to investigate a potential protective effect of endogenous IL‐33 during atherosclerosis associated with a cholesterol‐rich diet. The results described herein show that the deficiency of the endogenous IL‐33/ST2‐axis does not impact the development of atherosclerosis in ApoE‐deficient mice.

## Results and Discussion

### Deficiency of endogenous IL‐33 signaling does not affect the development of atherosclerosis

To examine the effect of endogenous IL‐33 signaling on the development of atherosclerosis, IL‐33^−/−^ApoE^−/−^, ST2^−/−^ApoE^−/−^, and ApoE^−/−^ mice were fed a cholesterol‐rich (1.25%, no cholate) diet. In addition, a blocking anti‐ST2 antibody or an isotype‐matched control antibody were used during feeding of the diet in ApoE^−/−^ mice (ApoE^−/−^, anti‐ST2 or ApoE^−/−^, isotype control). Efficacy of the blocking anti‐ST2 antibody was demonstrated previously 12, 13. At the beginning of the cholesterol‐rich diet, body weight was not different between the groups (Supporting Information Table S1), but there was some variation of serum total cholesterol levels between the groups. (Supporting Information Table S2). After 10 weeks of cholesterol‐rich diet, the body weight increased significantly, but without any significant difference between the groups (Supporting Information Table S1). The cholesterol‐rich diet induced a significant increase of serum total cholesterol in all groups with the exception of ST2^−/−^ApoE^−/−^ mice (Supporting Information Table S2). The cholesterol levels after 10 weeks of diet were significantly higher in IL‐33^−/−^ApoE−/ mice than in ST2^−/−^ApoE^−/−^ mice.

**Figure 1:**
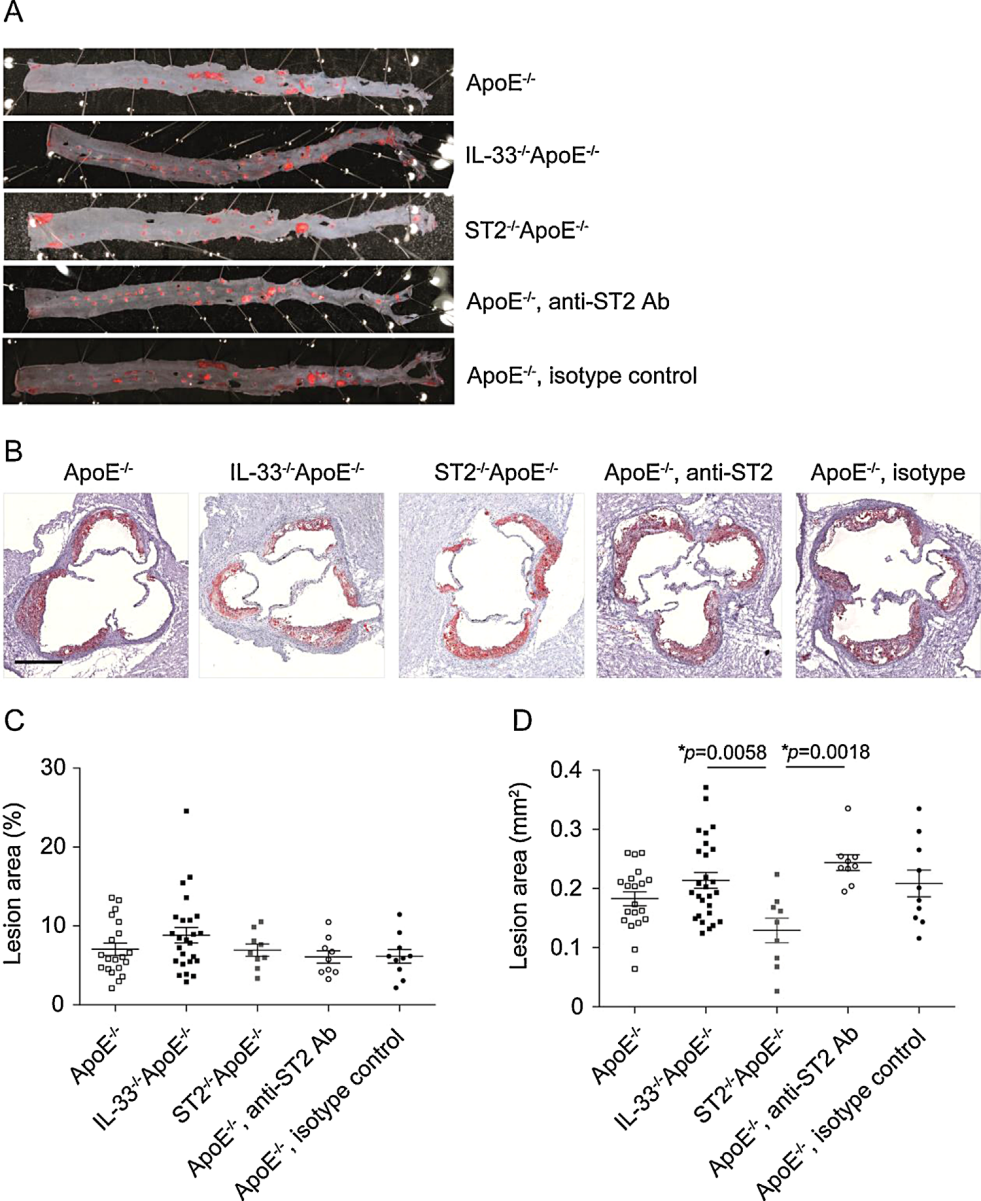
Development of atherosclerosis in mice deficient in IL‐33/ST2 signaling. Representative photographs (A, B) and quantitative analysis (C, D) of atherosclerotic lesion areas in thoracic‐abdominal aortas (A, C) and aortic sinuses (B, D) of ApoE^−/−^ (*n* = 20), IL‐33^−/−^ApoE^−/−^ (*n* = 25‐26), ST2^−/−^ApoE^−/−^ (*n* = 9), or ApoE^‐/‐^ mice injected with either a blocking anti‐ST2 (*n* = 9) or the isotype control antibody (*n* = 10) after 10 weeks of a cholesterol‐rich diet. All samples were stained with Oil Red O (red) for lipids and sections of aortic sinuses were counterstained with hematoxylin (purple). The extent of atherosclerotic lesions was analyzed by computer‐assisted image quantification as percentage of lipid deposition within the total surface area of the thoracic‐abdominal aortas (C) or as absolute values of lipid deposition in aortic sinuses (D), respectively. Data are shown as the mean ± SEM, 1‐way ANOVA, and Tukey's post‐test for pairwise comparison. Scale bar: 100 μm.

No significant differences in the extent of atherosclerotic lesions were observed in the thoracic‐abdominal aorta among all groups (Fig. 1A, C). The lesion area in the aortic sinus in IL‐33^−/−^ApoE^−/−^ mice was significantly increased by 66% (*P *= 0.0058) as compared to ST2^−/−^ApoE^−/−^ mice, but not different from the one in ApoE^−/−^ control mice (*P *= 0.4349; Fig. 1B, D). No difference in lesion area in the aortic sinus was detected after the treatment of ApoE^−/−^ mice with the anti‐ST2 antibody compared to the injection with an isotype control antibody (*P *= 0.7255) or to untreated ApoE^−/−^ mice (*P *= 0.1110). The lesion area in the aortic sinus in ApoE^−/−^ mice treated with the blocking anti‐ST2 antibody was significantly increased by 89% (*P *= 0.0018) as compared to ST2^−/−^ApoE^−/−^ mice.

Our results differ from those of Miller et al., who showed that systemically administered IL‐33 reduced the development of atherosclerosis in ApoE^−/−^ mice and that the administration of soluble ST2, which neutralizes endogenous IL‐33 by acting as a decoy receptor, exacerbated the development of atherosclerotic plaques in the aortic sinus 11. The discrepant results might be explained by different experimental conditions, such as the composition and the duration of the diet, as well as the sanitary status of the animal housing, which are all variables with an impact on the development of atherosclerosis 14, 15. In contrast to Miller et al., who fed a high‐fat diet with low cholesterol (21% lard, 0.15% cholesterol) for 12 weeks to mice maintained in a pathogen‐free facility, we fed the mice a high‐fat diet with high cholesterol (20% fat [40 kcal%], 1.25% cholesterol) for 10 weeks under conventional conditions. We can, thus, speculate that different housing conditions with a different microbiota and/or a prolonged duration of diet might influence the experimental results.

Another important difference was the use by Miller et al. of systemic administration of IL‐33, which is known to induce Th2 responses 2, 16, a situation which may not necessarily reflect the effects of physiological amounts of endogenous IL‐33. Of course, the administration of soluble ST2 should be mimicking IL‐33 deficiency. However, we cannot exclude that soluble ST2 binds other proteins or exerts other activities in vivo 17.

Unexpectedly, we also observed a different phenotype in ST2^−/−^ApoE^−/−^ as compared to IL‐33^−/−^ApoE^−/−^ mice. Indeed, with ST2 being the receptor for IL‐33, one would expect similar phenotypes in ST2^−/−^ApoE^−/−^ and IL‐33^−/−^ApoE^−/−^ mice. However, we recently published other discrepant findings between IL‐33^−/−^ and ST2^−/−^ mice in K/BxN serum‐induced arthritis, with ST2^−/−^ mice showing a reduced incidence and severity of arthritis compared to both WT and IL‐33^−/−^ mice 18. Furthermore, discrepant findings in studies using different ST2^−/−^ lines, in different studies using the same ST2^‐/‐^ lines, or between IL‐33^−/−^ and ST2^−/−^ mice have already been described by others, especially in the context of allergic airway inflammation 19, 20, 21, 22. In order to explain the discrepant observations between the IL‐33^−/−^ and ST2^−/−^ mice observed in K/BxN serum‐induced arthritis, we investigated a number of potential confounding variables. We did not detect any significant impact of ST2 gene targeting on the expression and functionality of other IL‐1R family members, nor the expression of miRNA in the vicinity of the ST2 locus. Another possible explanation might lie in differences in genetic background between the IL‐33^−/−^ApoE^−/−^ and ST2^−/−^ApoE^−/−^ mouse lines. While the IL‐33^−/−^ mouse line was originally backcrossed for more than 10 generations into the C57BL/6 background, the backcross of the ST2^−/−^ mouse line is less complete with remnants of genetic material from the original 129 mouse strain 18. Of note, ApoE^−/−^ mice in a 129 background have smaller lesions in the aortic roots 23 and the aorta 24 than ApoE^−/−^ mice in a C57BL/6 background. Another possibility could be that ST2 has other ligands than IL‐33 or that IL‐33 has unique intracellular activities independent of ST2.

In order to investigate the role of ST2 using a different approach, we thus injected ApoE^−/−^ mice with a neutralizing anti‐ST2 or an isotype matched control antibody during the entire period of the cholesterol‐rich diet. Atherosclerotic lesions in the aortic sinus of ApoE^−/−^ mice treated with the neutralizing anti‐ST2 antibody did not significantly differ from those of ApoE^−/−^ mice treated with the isotype control antibody. This observation and the additional finding that serum total cholesterol significantly increased after 10 weeks on a cholesterol‐rich diet in all groups but not in ST2^−/−^ApoE^−/−^ mice suggest that results obtained with the ST2^−/−^ApoE^−/−^ mice should be interpreted with caution.

### Expression of IL‐33 in cells of the adventitia

**Figure 2:**
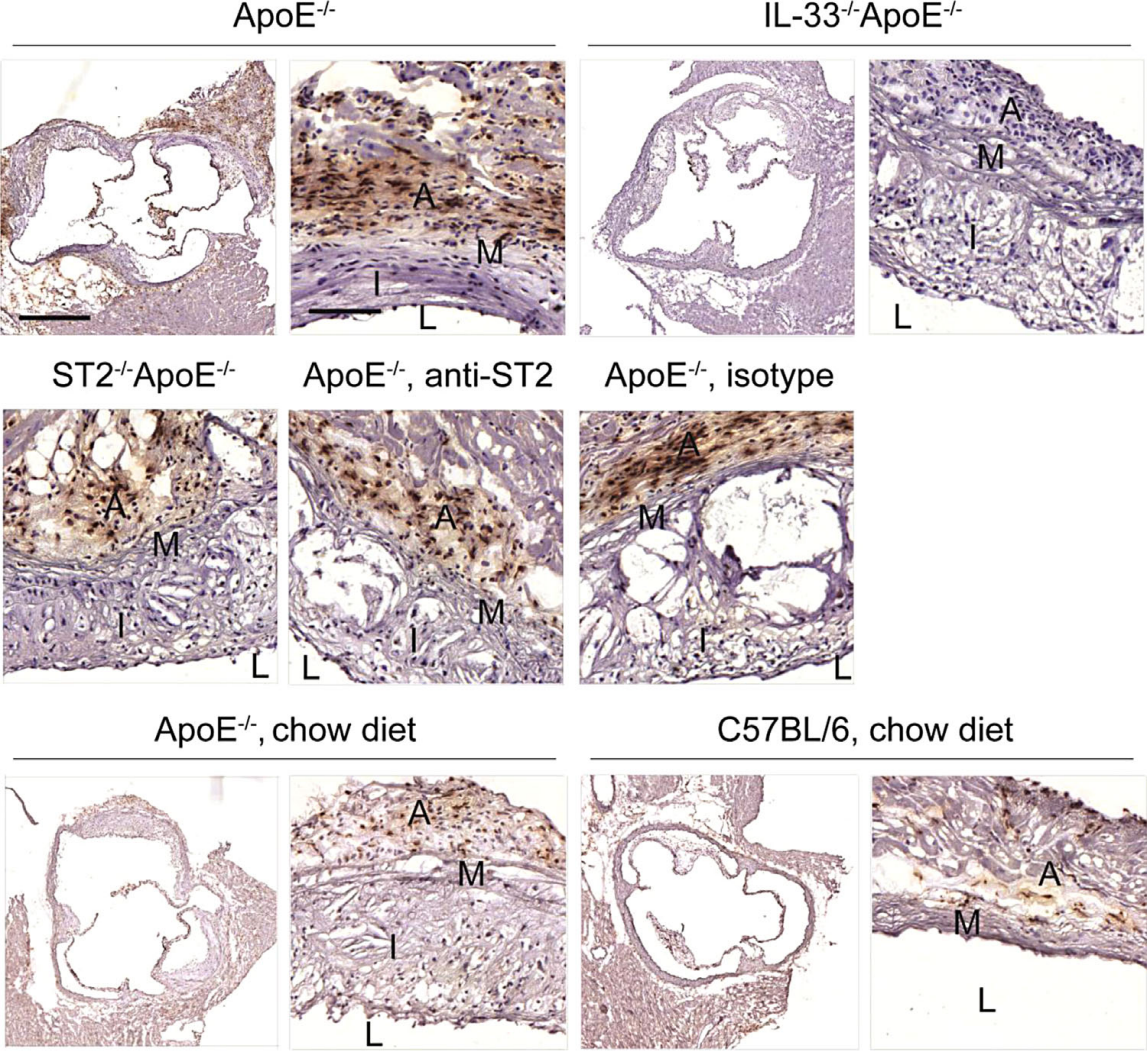
Expression of IL‐33 in aortic sinuses. Representative immunohistochemical localization of IL‐33 on sections of aortic sinuses of ApoE^−/−^ (cholesterol‐rich diet or chow diet), IL‐33^−/−^ApoE^−/−^, ST2^−/−^ApoE^−/−^, or ApoE^−/−^ mice injected with either a blocking anti‐ST2 or the isotype control antibody (cholesterol‐rich diet) and C57BL/6 control mice (chow diet). Scale bars: 500 μm for the overview, 100 μm for the zoom. A, adventitia; M, media; I, Intima; L, lumen.

IL‐33 expression has been reported in cells expressing α‐smooth muscle actin in the adventitia of atherosclerotic aortas 11. Herein, we confirm the expression of IL‐33 in the nucleus of cells in the adventitia of all groups except for IL‐33^−/−^ApoE^−/−^ mice (Fig 2, upper and middle panels). Less IL‐33‐expressing cells are detected in the aortic sinus of ApoE^−/−^ and C57BL/6 mice fed a chow diet (Fig. 2, lower panels), suggesting that IL‐33 expression is either increased in resident cells in the adventitia during atherosclerosis and/or IL‐33‐expressing cells are recruited to the adventitia.

### The deficiency of endogenous IL‐33 signaling has no impact on the Th1/Th2 cytokine profile

**Figure 3:**
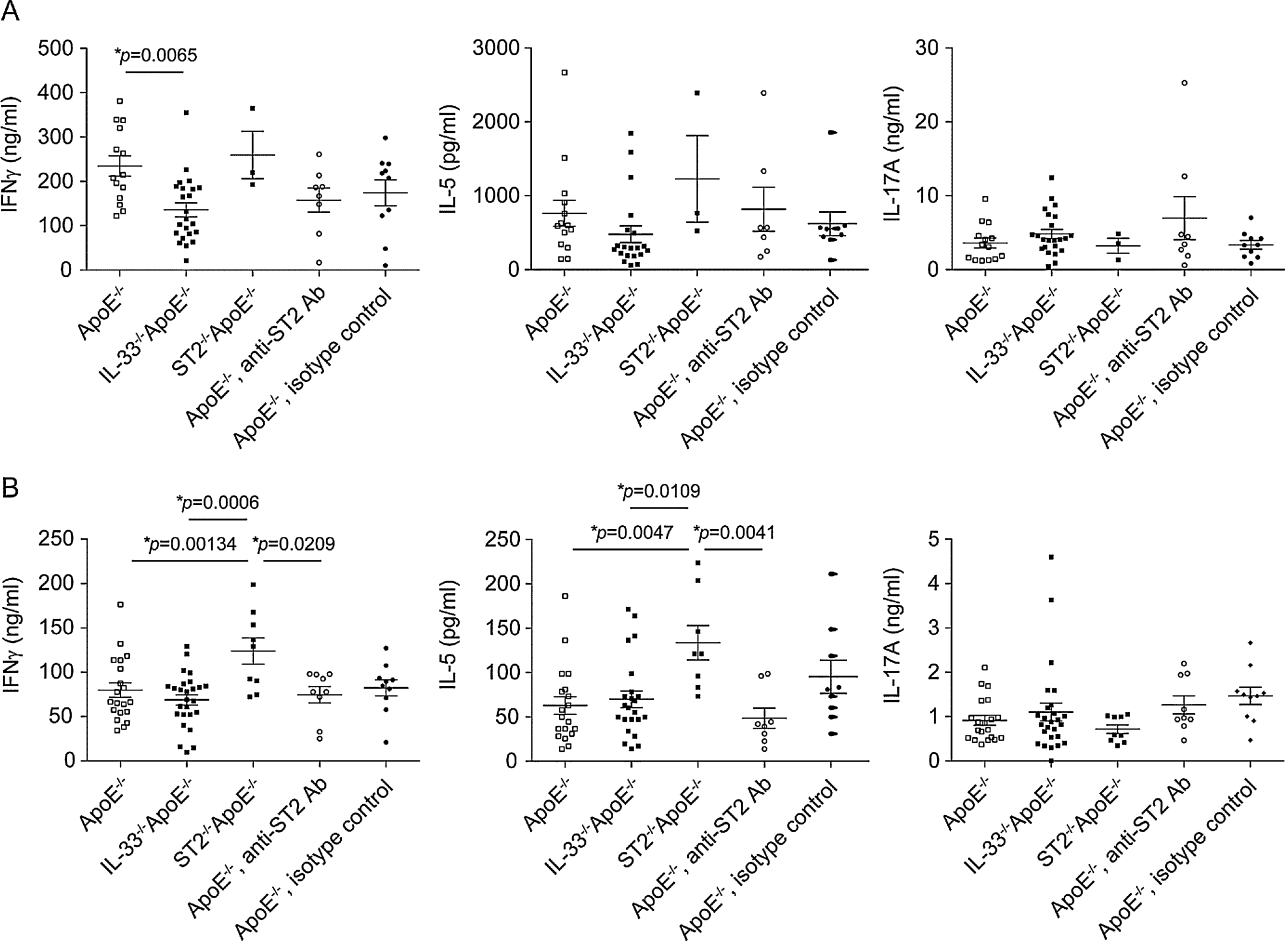
The impact of the deficiency of endogenous IL‐33 signaling on the Th1/Th2 cytokine profile in lymph node cells. Cytokine expression profile of lymph node cells after 10 weeks of cholesterol‐rich diet. Mediastinal (A) and peripheral (B) lymph node cells from ApoE^−/−^ (*n* = 14/*n* = 19–20), IL‐33^−/−^ApoE^−/−^(*n* = 20‐23/*n* = 23–26), ST2^−/−^ApoE^−/−^(*n* = 3/*n* = 8–9), or ApoE^‐/‐^ mice injected with either a blocking anti‐ST2 (*n* = 7–9/*n* = 8–9) or the isotype control antibody (*n* = 9–10/*n* = 9–10) were stimulated with anti‐CD3 and anti‐CD28 for 72 h. IFNγ, IL‐5, and IL‐17A protein levels were measured by ELISA in the supernatant. Data are shown as the mean ± SEM, 1‐way ANOVA, and Tukey's post‐test for pairwise comparison.

To determine whether the absence of IL‐33 or ST2 promotes a Th1 rather than a Th2 immunological profile as described 11, cytokine responses were assessed in in vitro cultivated sinus‐draining mediastinal and peripheral (including axillary and inguinal) lymph node cells. Levels of IFNγ were lower in the supernatant of mediastinal (Fig. 3A), but not peripheral (Fig. 3B) lymph node cells from IL‐33^−/−^ApoE^−/−^ mice compared to ApoE^−/−^ controls, a difference which is not reflected by the lesion areas. Therefore, the relevance of this difference in the cytokine production remains arguable. Peripheral lymph node cells from ST2^−/−^ApoE^−/−^ mice secreted significantly higher levels of both the Th1 cytokine IFNγ and the Th2 cytokine IL‐5 compared to lymph node cells from ApoE^−/−^, IL‐33^−/−^ApoE^−/−^, and ApoE^−/−^ mice which received the neutralizing anti‐ST2 antibody. We examined also the levels of IL‐17A, since an increased concentration of IL‐17 has been reported in plasma of unstable angina and acute myocardial infarction patients, suggesting a potential role of IL‐17 in atherosclerosis 25. However, IL‐33/ST2 axis deficiency was devoid of any impact on IL‐17A secretion. Taken together, the deficiency or blockage of IL‐33 or ST2 did not cause major and consistent changes concerning the Th1/Th2 profile.

### Concluding remarks

The results presented herein do not support a role of endogenously expressed IL‐33 and ST2 in atherogenesis in ApoE‐deficient mice.

## Materials and Methods

### Mice

C57BL/6J Apolipoprotein E‐deficient (ApoE^−/−^; *Apoe* ^tm1Unc^) mice were obtained from Jackson Laboratory and crossed with C57BL/6 IL‐33^−/−^ mice (B6.129Sv‐*Il33*) obtained from Amgen, Inc. (Thousand Oaks, CA) 18 or C57BL/6 ST2^‐/‐^ mice (Il1rl1^tm1Anjm^) obtained from the MRC Laboratory of Molecular Biology (Cambridge, UK) 26 in order to generate IL‐33^−/−^ApoE^−/−^ or ST2^−/−^ApoE^−/−^ lines, respectively. Genotyping was performed by PCR by following Jackson Laboratory protocol for ApoE^−/−^ mice or as previously described for IL‐33^−/−^ 18. Genotyping for the *St2* gene was performed by a 3‐primer PCR combining a common reverse primer (5'‐GGAAATGCAACCAGAAGTGCACAGG‐3') with forward primers specific for the wild type (5'‐GCTGGATAAAGCTATATCATGG‐3') or the KO (5'‐GATTGCACGCAGGTTCTC‐3') alleles. All mice were maintained under conventional conditions in the animal facility of the Geneva University School of Medicine, and water and food were provided ad libitum. Animal studies were approved by the Animal Ethics Committee of the Geneva Veterinarian Office (licence number: 1067/3620/1) and were performed according to the appropriate codes of practice.

### Biological reagents

The monoclonal murinized IgG1 blocking anti‐ST2 and the isotype matched control antibodies were generated at Amgen, Inc. Efficacy of the blocking anti‐ST2 antibody was demonstrated previously 12, 13. Cell culture media were obtained from Invitrogen Life Technologies (Basel, Switzerland).

### Experimental design

Male ApoE^−/−^, IL‐33^−/−^ApoE^−/−^, and ST2^−/−^ApoE^−/−^ mice were used at 10 weeks of age and then placed on a cholesterol‐rich diet (20% fat [40 kcal%], 1.25% cholesterol, no cholate; Research Diets, New Brunswick, NJ) for 10 weeks. Male ApoE^−/−^ and C57BL/6 mice fed a chow diet for a total of 20 weeks served as controls. In a separate experiment, male ApoE^−/−^ mice were randomly grouped at 10 weeks of age and injected twice per week intraperitoneally with PBS, 200 μg anti‐ST2, or 200 μg isotype control antibody for 10 weeks during the feeding of the cholesterol‐rich diet. As controls, male IL‐33^−/−^ApoE^−/−^ mice were injected intraperitoneally twice per week for 10 weeks with PBS. Mice were weighed before and after diet. Peripheral blood was collected before and after diet. At 20 weeks of age aortas were separated into two parts, of which the thoracic‐abdominal parts were fixed in 2% paraformaldehyde and the aortic sinuses were snap‐frozen in OCT compound. In order to compare the data obtained in the two independent experiments, the values of the lesion areas in the thoracic‐abdominal aorta and the aortic sinus of not treated and PBS‐injected ApoE^−/−^ mice from the first and second experiment, respectively, were compared by unpaired two‐tailed Student's *t*‐test. Since there was no significant difference, the two experiments were pooled.

### Oil Red O staining and atherosclerotic lesion analysis

The extent of atherosclerosis was assessed in thoracic‐abdominal aortas and aortic sinus cryosections (7 μm) with Oil Red O staining. The fixed thoracic‐abdominal aortas were stained with Oil Red O solution (58% isopropanol, 0.2% Oil Red O; Sigma–Aldrich, Buchs, Switzerland) overnight at 4°C and opened longitudinally to the iliac bifurcation. The percentage of lipid deposition (red staining) in the thoracic‐abdominal aortas was calculated within the total surface area using the Definiens Developer XD Software. Frozen aortic sinus sections were dried at RT, fixed in 10% formalin, rinsed with distilled H_2_O, and then with 60% isopropanol. The sections were incubated with Oil Red O solution (60% isopropanol, 0.3% Oil Red O) for 15 min at RT, rinsed with 60% isopropanol, and counterstained with hematoxylin. Slides were scanned with Mirax Scan (Carl Zeiss, Jena, Germany). For the quantification of atherosclerotic lesions in the sinuses, the average of the lesion area from 5 sections distant by 35 μm from each other was calculated by measuring the absolute area of lipid deposition using Definiens Developer XD Software. Pictures were taken using the Pannoramic Viewer software (3D HISTECH, Budapest, Hungary).

### Serum analysis

Mouse serum total cholesterol concentrations were measured as described 27.

### Immunohistochemistry

IL‐33 expression was examined on frozen aortic sinus sections (7 μm). In brief, after drying at 37°C, rehydration in PBS and fixation in 4% paraformaldehyde, endogenous peroxidase activity was blocked and the sections were incubated with a polyclonal goat anti‐mouse IL‐33 antibody (R&D Systems, Vienna, Austria; at 1 μg/mL) as described 18 and counterstained with hematoxylin. Slides were scanned with Mirax Scan (Carl Zeiss) and pictures taken using the Pannoramic Viewer software (3D HISTECH).

### Culture of lymph node cells

Lymph node cells were removed and 1 × 10^6^ cells/mL were activated with plate‐bound anti‐mouse CD3 and anti‐mouse CD28 (both 2 μg/mL; BD Pharmingen, Allschwil, Switzerland) in RPMI, 10% FCS, 50 μM β‐ME, 100 U/mL penicillin, and 100 μg/mL streptomycin for 72 h.

## ELISA

IFN‐γ, IL‐5, and IL‐17A protein levels in lymph node cell supernatants were determined using ELISA Ready Set Go kits (IFN‐γ, IL‐17A) from eBioscience (Vienna, Austria) or a DuoSet ELISA kit (IL‐5) from R&D Systems.

### Statistical analysis

Significant variations were calculated using the unpaired or paired two‐tailed Student's *t*‐test or 1‐way ANOVA with Tukey's post‐test for pairwise comparison when the ANOVA was statistically significant, as indicated in the figure legends. *P *< 0.05 was considered significant. Results are expressed as the mean ± SEM.

## Author Contributions

PM designed the study, performed experiments, analyzed the data, and wrote the manuscript. GP analyzed data and wrote the manuscript. ER and EW performed experiments and analyzed the data. IM performed experiments and analyzed the data. RWJ analyzed data and wrote the manuscript. DES and BRK analyzed the data and wrote the manuscript. CG supervised the project, designed the study, analyzed the data and wrote the manuscript.

### Conflict of Interest

None declared.

## Supporting information

Additional supporting information may be found in the online version of this article at the publisher's web‐site.

**Table S1**: Body weight (g) before and after cholesterol‐rich diet.
**Table S2**: Serum total cholesterol concentrations (mM) before and after cholesterol‐rich diet.
