# Supplementary material for: Atherosclerosis severity is not affected by a deficiency in IL‐33/ST2 signaling
Source: Immun Inflamm Dis. 2015 May 10;3(3):239–46. doi: 10.1002/iid3.62 (PMC4578523; doi:10.1002/iid3.62)
Supplement: Supplementary file 1 — Table S1: Body weight (g) before and after cholesterol‐rich diet. Table S2: Serum total cholesterol concentrations (mM) before and after cholesterol‐rich diet. [file IID3-3-239-s001.pdf]

Supporting Information Table 1: Body weight (g) before and after cholesterol-rich.

Values represent the mean ± SEM. *p<0.001, significantly different before and after feeding 10 weeks a cholesterol-rich diet as analyzed by paired two-tailed Student's t-test. Not significantly different between groups before or after diet as analyzed by one-way ANOVA and Tukey's post-test for pairwise comparison.

	$ApoE^{-/-}$ (n=19)	$IL\text{-}33^{-/-}ApoE^{-/-}$ (n=28)	$ST2^{-/-}ApoE^{-/-}$ (n=9)	$ApoE^{-/-}$, anti-ST2 (n=9)	$ApoE^{-/-}$, isotype (n=10)
before diet	26.33 ± 0.55	27.15 ± 0.47	27.17 ± 0.55	25.88 ± 0.77	26.03 ± 0.54
after diet	32.07 ± 0.52*	31.41 ± 0.55*	30.24 ± 0.78*	29.75 ± 1.20*	30.21 ± 0.77*

Supporting Information Table 2: Serum total cholesterol concentrations (mM) before and after cholesterol-rich diet.

Values represent the mean ± SEM. *p<0.05, significantly different before and after feeding 10 weeks a cholesterol-rich diet as analyzed by paired two-tailed Student's t-test. §, *p*=0.016 vs. ApoE$^{-/-}$, anti-ST2; #, *p*=0.0032 vs. ApoE$^{-/-}$, anti-ST2 or *p*=0.022 vs. ApoE$^{-/-}$, isotype; †, *p*=0.032 vs. ST2$^{-/-}$ApoE$^{-/-}$, one-way ANOVA and Tukey's post-test for pairwise comparison.

	ApoE$^{-/-}$ (n=20)	IL-33$^{-/-}$ApoE$^{-/-}$ (n=28)	ST2$^{-/-}$ApoE$^{-/-}$ (n=9)	ApoE$^{-/-}$, anti-ST2 (n=9)	ApoE$^{-/-}$, isotype (n=10)
before diet	6.83 ± 0.31	6.23 ± 0.21§	5.59 ± 0.17#	7.69 ± 0.41	7.29 ± 0.43
after diet	9.15 ± 0.45*	9.96 ± 0.67*,†	6.76 ± 0.73	9.93 ± 0.82*	10.33 ± 0.78*
